# Phytochemical Profile and In Vitro Antioxidant, Antimicrobial, Vital Physiological Enzymes Inhibitory and Cytotoxic Effects of *Artemisia jordanica* Leaves Essential Oil from Palestine

**DOI:** 10.3390/molecules26092831

**Published:** 2021-05-10

**Authors:** Nidal Jaradat

**Affiliations:** Department of Pharmacy, Faculty of Medicine and Health Sciences, An-Najah National University, P.O. Box 7, Nablus 00970, Palestine; nidaljaradat@najah.edu

**Keywords:** *Artemisia jordanica*, essential oil, antioxidant, anti-obesity, antidiabetic, antimicrobial, anti-inflammatory, cytotoxicity

## Abstract

*Artemisia jordanica* (AJ) is one of the folkloric medicinal plants and grows in the arid condition used by Palestinian Bedouins in the Al-Naqab desert for the treatment of diabetes and gastrointestinal infections. The current investigation aimed, for the first time, to characterize the (AJ) essential oil (EO) components and evaluate EO’s antioxidant, anti-obesity, antidiabetic, antimicrobial, anti-inflammatory, and cytotoxic activities. The gas chromatography-mass spectrometer (GC-MS) technique was utilized to characterize the chemical ingredients of (AJ) EO, while validated biochemical approaches were utilized to evaluate the antioxidant, anti-obesity and antidiabetic. The microbicidal efficacy of (AJ) EO was measured utilizing the broth microdilution assay. Besides, the cytotoxic activity was estimated utilizing the (MTS) procedure. Finally, the anti-inflammatory activity was measured utilizing a COX inhibitory screening test kit. The analytical investigation revealed the presence of 19 molecules in the (AJ) EO. Oxygenated terpenoids, including bornyl acetate (63.40%) and endo-borneol (17.75%) presented as major components of the (AJ) EO. The EO exhibited potent antioxidant activity compared with Trolox, while it showed a weak anti-lipase effect compared with orlistat. In addition, the tested EO displayed a potent α-amylase suppressing effect compared with the positive control acarbose. Notably, the (AJ) EO exhibited strong α-glucosidase inhibitory potential compared with the positive control acarbose. The EO had has a cytotoxic effect against all the screened tumor cells. In fact, (AJ) EO showed potent antimicrobial properties. Besides, the EO inhibited the enzymes COX-1 and COX-2, compared with the anti-inflammatory drug ketoprofen. The (AJ) EO has strong antioxidant, antibacterial, antifungal, anti-α-amylase, anti-α-glucosidase, and COX inhibitory effects which could be a favorite candidate for the treatment of various neurodegenerative diseases caused by harmful free radicals, microbial resistance, diabetes, and inflammations. Further in-depth investigations are urgently crucial to explore the importance of such medicinal plants in pharmaceutical production.

## 1. Introduction

For centuries, humankind has utilized various plant species for many medical, nutraceutical, flavor, food additive, and cosmetics applications. Medicinal herbs are in high demand for primary health care in many countries of the developing and developed world because they have fewer adverse effects, are safer, and some of them are more effective than synthetic medications [1]. Many plants contain extractable secondary metabolic compounds with wide biological activity and many of the recently used pharmaceutical formulations are isolated from plants [2].

Essential oils (EOs) are combinations of various aromatic volatile secondary metabolic molecules that are mainly isolated from plants. They have various biologicals effects including antioxidant, antimicrobial, anticancer, digestive enzyme inhibitory, anti-inflammatory, anthelmintic, and many others [3]. Commercially, EOs are used in the manufacturing of hygienic products, food flavorings, beverages, household products, soaps, pharmaceuticals, perfumes, cosmetics, and other products [4].

These days, EOs have been thoroughly investigated as potential antioxidant agents to replace synthetic ones. In fact, EOs are mixtures of oxygenated terpenoids, monoterpene, sesquiterpenes, phenols, and other molecules. In most cases, the antioxidant activity of the EOs can be easily explained by the presence of oxygenated hydrocarbons such as sesquiterpenoids and monoterpenoids in their composition which are well-known as potent antioxidants and able to trap the chain-carrying lipid peroxyl radicals responsible for lipid oxidation. Recently, it has been reported that other simple components of EOs including terpenoids may potentially contribute to the scavenging of the harmful free radicals [5].

De facto, many studies reported that the mechanism of action of the EOs as anti-obesity agents may be attributed to several possible factors such as their ability to suppress the effect of pancreatic lipase enzyme, increase the glycerol level in the plasma, or inhibit the accumulation of fats in the human body [6,7].

Recently, diabetes is recognized as a global problem, with the number of diabetics rising in 1980 from 200 million to 420 million in 2014. In the previous two years, it was the leading cause of million deaths worldwide, according to WHO, it was ranked as the seventh leading cause of death [8,9,10].

Several in vivo and in vitro investigations proved that the natural plants’ EOs reduced blood glucose levels by improving the distorted pancreatic β-cells architecture of diabetic animals or by inactivation of enzymes such as α-glucosidase and α-amylase that are responsible for the metabolism of carbohydrates in the gastrointestinal tract. Overall, the antidiabetic effect of the EOs may prove to be of clinical value in the treatment of hyperglycemia [11,12,13,14].

Natural plant EOs have been reported to possess antiproliferative, antioxidant, antimutagenic, and detoxifying activities, as well as they, have cancer preventative properties. In addition, EOs can increase the efficacy of many chemotherapeutic agents such as docetaxel and paclitaxel also may improve the immune system function for patients suffering from cancer. However, several investigations revealed that EOs have anticancer activity throughout several mechanisms of actions including that they can slow cancer cell division by interfering with the cell cycle, induce apoptosis, inhibit phase 1 enzymes that convert harmless compounds into carcinogens, and induce phase 2 enzymes that can attach carcinogens to compounds that facilitate speedy excretion [15].

Actually, EOs inhibit or slow the microbial growth through a variety of targets, including their ability to destroy the microbial cells membrane s and cytoplasm, and in some cases, EOs can change the morphology of some microbial species [16].

Recently, due to the extreme negative cardiovascular and gastrointestinal effects of non-steroidal anti-inflammatory drugs (NSAIDs) and selective COX-2 inhibitors, researchers are looking for more potential anti-inflammatory agents with fewer adverse effects than NSAIDs [17]. As a result, EOs are now seen as promising targets for the next wave of anti-inflammatory treatments as well as they can inhibit transcription of NF-κB and suppress the cascade of arachidonic acid [18,19].

In folk medicine all over the world, Artemisia species are widely utilized as antioxidant, antifungal, antimalarial, insecticidal, antimicrobial, antispasmodic, antitumor, and anti-inflammatory activities [20].

*Artemisia jordanica* Danin (AJ); is a perennial aromatic herbaceous shrubby plant that belongs to the Compositae family with succulent, narrow, and simple leaves. Its native range is South Palestine to Iraq. The leaves decoction of (AJ) plant is utilized in traditional Bedouin medicine as an antihypertensive, antispasmodic, and anthelmintic medicine [21].

As far as I know, no investigation has yet identified the chemical components and examined the biological effects of EOs obtained from the (AJ) aromatic plant. The present work aims to identify and quantify the chemical ingredients of (AJ) EO for the first time and evaluate its antioxidant, anti-obesity, antidiabetic, antimicrobial, anti-inflammatory, and cytotoxic activities.

## 2. Results

### 2.1. Phytochemistry

**Figure 1:**
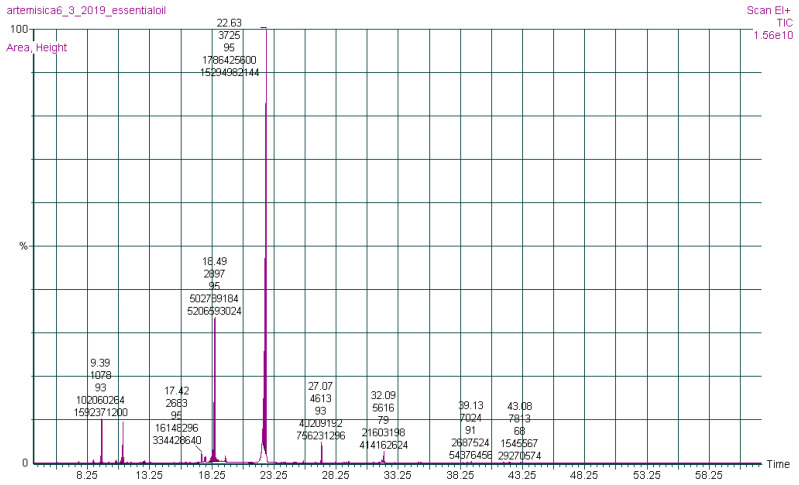
Gas chromatography-mass spectrometry chromatograph of *Artemisia jordanica* essential oil.

The chemical constituents of the (AJ) EO were investigated by gas chromatography-mass spectrometry (GC-MS) analysis (Figure 1). Nineteen compounds were qualitatively and quantitatively characterized in the EO of the (AJ) leaves, representing 100% of the total EO mass, as presented in Table 1, where bornyl acetate (63.40%) and endo-borneol (17.75%) were identified as the abundant ingredients. Moreover, the major phytochemical classes were oxygenated monoterpenoids (85.98%) and oxygenated sesquiterpenoid (8.01%).

### 2.2. Antioxidant Activity

**Figure 2:**
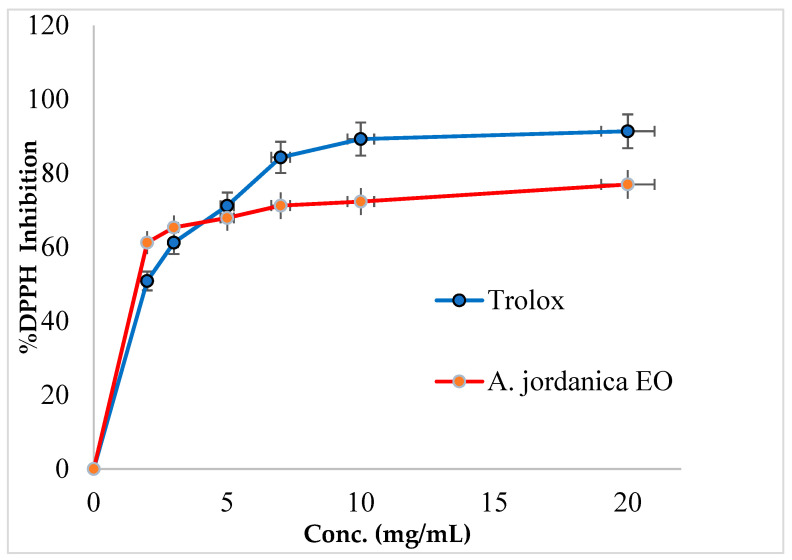
DPPH inhibitory activity by *Artemisia jordanica* essential oil and Trolox.

In this study, the (AJ) EO showed a dose-dependent inhibitory activity against DPPH free radical activity and has 74.88% of the antioxidant potential compared with a standard antioxidant compound Trolox. The DPPH inhibitory activity by (AJ) EO and Trolox is shown in Figure 2 and the IC_50_ values are presented in Table 2.

### 2.3. Target Metabolic Enzyme Inhibitory Activity

**Figure 3:**
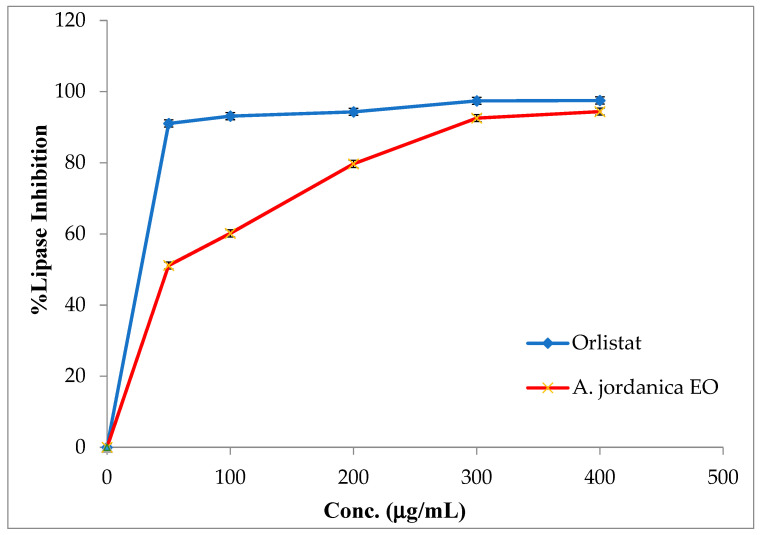
Porcine pancreatic lipase inhibitory activity by *Artemisia jordanica* essential oil and orlistat.

**Figure 4:**
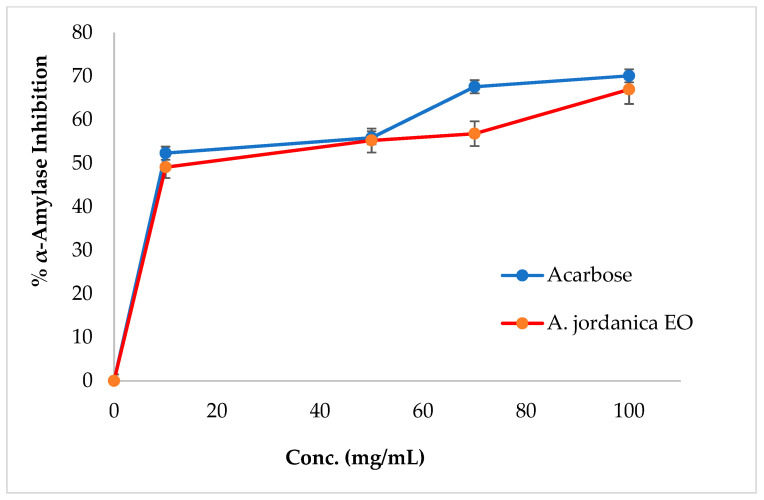
α-Amylase inhibitory activity by *Artemisia jordanica* essential oil and acarbose.

**Figure 5:**
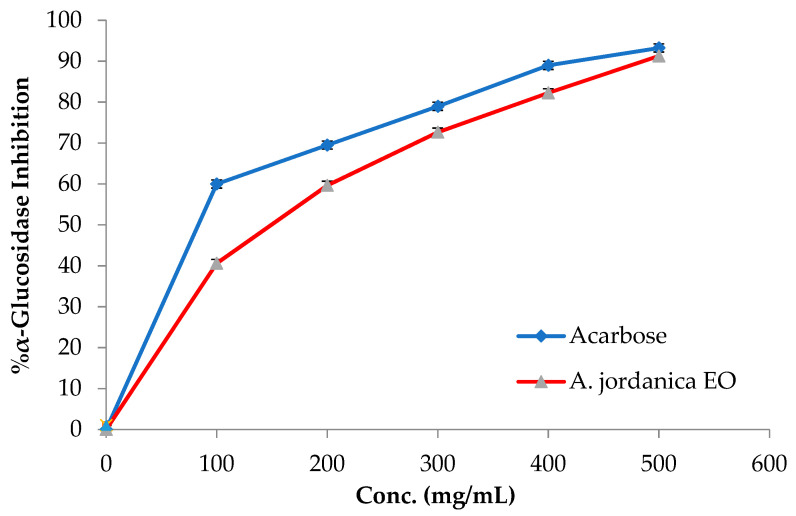
α-Glucosidase inhibitory activity by *Artemisia jordanica* essential oil and acarbose.

The EO of the (AJ) plant showed dose-dependent inhibitory activity against porcine pancreatic lipase, α-amylase, and α-glucosidase, compared with the positive controls, which were the anti-obesity drug orlistat and the antidiabetic medication acarbose. The results arising from the lipase, α-amylase, and α-glucosidase inhibitory activities evaluations of the (AJ) EO are shown in Figure 3, Figure 4 and Figure 5, while the IC_50_ values are given in Table 2.

### 2.4. Cytotoxicity

**Figure 6:**
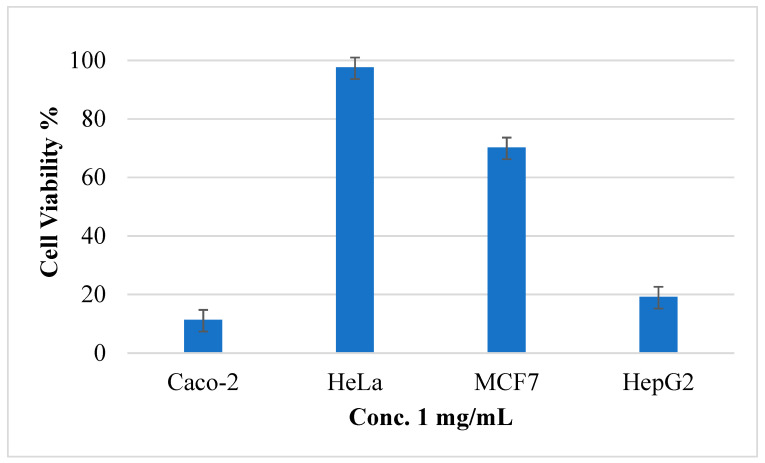
The cell viability percentage of *Artemisia jordanica* essential oil against four cancer cell lines at 1 mg/mL concentration ± SD.

After treatment of HeLa, MCF-7, Caco-2, and Hep3B tumor cells with five different concentrations of (AJ) EO, the MTS assay results showed that the EO has cytotoxic activity against all the screened tumor cells as presented in Table 2. However, the cell viability percentage of the AJ EOs was calculated against all cancer cell at concentration 1 mg/mL and presented in Figure 6. It was clear that the EOs has potent cytotoxic activities against Caco-2 and hepG2 with cell viability percentage 11.33% and 19.19%, respectively. In contrast the cell viability percentage was high at this concentration against HeLa and MCF-7.

### 2.5. Antimicrobial Effect

The antimicrobial activity of (AJ) EO was established using the broth microdilution method. The (AJ) EO inhibited the growth of most of the tested microbial strains. Table 3 depicts that (AJ) EO has remarkable antimicrobial effects against MRSA, *S. aureus*, *P. vulgaris*, and *C. albicans* compared with the positive antimicrobial controls, the commercial antibiotics ciprofloxacin and ampicillin, and commercial antifungal drug fluconazole, while the *P. aeruginosa* and *E.* *coli* strains were resistant to (AJ) EO.

### 2.6. COX Inhibitory Activity

**Figure 7:**
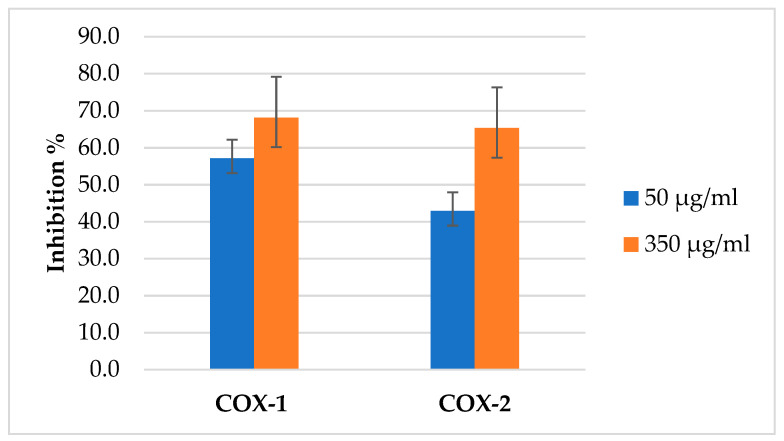
The inhibition percentage of *Artemisia jordanica* essential oil against COX1 and COX2 at two different concentrations 50 and 350 µg/mL ± SD.

The (AJ) EO was evaluated against COX enzymes, and its activity was compared with the positive control, the commercial NSAID Ketoprofen. In two concentrations 50 and 350 µg/mL, the percentage inhibition of COX-1 and COX-2 increased with an increase in the concentration of EO used as presented in Figure 7. The (AJ) EO showed potential inhibitory activity towards COX-1 and COX-2 enzymes as presented in Table 4.

## 3. Discussion

Since the beginning of history, medicinal herbal products were a valuable gift from nature for the treatment and prophylaxis of lethal diseases to humankind and animals. One hundred years ago, a simple wound could cause gangrenous and death, but now such as this infection can be treated quickly with an antibiotic ointment such as fusidic acid. Therefore, scientists must not lose the hope of discovering effective treatments for incurable diseases such as cancer, Alzheimer’s, diabetes, hypertension, and many others.

In nature, one of the main roles of the plants’ secondary metabolites including EOs is a protection function against bacterial, viral, fungal, insects, and herbivore animal attacks. They are also able to attract certain kinds of insects for the pollination process. Thereby, EOs can play potential antimicrobial activity against various infectious diseases, flavoring agents in food industries, and main ingredients in cosmetics and perfumes [23]. The presence of heteroatomic compounds in the EOs can induce various biological activities such as antioxidant activity. In addition, oxygen-containing moieties such as oxygenated monoterpenoids and sesquiterpenoids have antioxidant activity more potent than nitrogen-containing structures such as aniline [24]. However, bornyl acetate as the main component of this EO may induce different pharmacological activities such as antioxidant and enzyme inhibitory activities. Among the EO compounds, the aromatic containing compounds such as camphene, sabinene, 2,3-dehydro-1,8-cineole, cineole, camphor, and nirol oxide have interesting antimicrobial activities as well as the aliphatic components, such as geranyl isovalerate, and Terpinen-4-ol showed potent antibacterial activities [25]. The oxygenated terpenoid compounds in high percentage of (AJ) EO such as endo-borneol and bornyl acetate have a high ability to penetrate the lipophilic lipids of the mitochondria and cytoplasmic membrane as well as they could disturb the structures and resulting in leakage of bacterial cell contents.

### 3.1. Phytochemical Constituents

The chemical ingredients of EOs depend on the plant’s species, climatic conditions, origin, and seasonal variations. The phytochemical composition of (AJ) EO was identified and quantified utilizing GC-MS; 19 molecules were identified in the screened EO, representing 100% of the total mass. The results revealed that (AJ) EO is mainly composed of oxygenated monoterpenoid, monoterpene hydrocarbon, sesquiterpene hydrocarbon, and oxygenated sesquiterpenoid phytochemical classes which accounted for 85.98%, 4.28%, 1.66%, and 8.01%, respectively. The major identified components were bornyl acetate (63.40%), endo-borneol (17.75%), and geranyl isovalerate (7.67%).

The current investigation characterized for the first time the compositions of (AJ) EO and found that the abundant molecule bornyl acetate which also was the major component of *Artemisia absinthium* L. EO from Cuba and presented 23.02% of the identified components [26].

However, bornyl acetate was one of the constituents but not the major one of various *Artemisia* species including *A. herba* [27], *A. frigida*, *A. argyrophylla* [28], *A. selengensis* [20], and *A. dracunculus* [29].

### 3.2. Antioxidant Activity

The in vitro DPPH free radical scavenging assay is intended to mimic the oxidation-reduction reactions that usually occur in living organisms, and it was utilized to evaluate the antioxidant properties of various kinds of biological and chemical samples. Regarding the antioxidant activity, the (AJ) EO was able to reduce DPPH radicals into the natural DPPH-H form, and this effect occurred in a dose-dependent manner. In fact, (AJ) EO exhibited potent antioxidant activity, with an IC_50_ value of 2.18 ± 0.24 µg/mL and 74.88% antioxidant potential compared with Trolox, which has an antioxidant IC_50_ dose of 1.58 ± 1.49 µg/mL. In fact, Trolox is a vitamin E analog with powerful antioxidant properties, and it is used in different biochemical or biological applications to prevent the production of harmful free radicals and to decrease the damage caused by oxidative stress.

A study conducted by Juteau et al. found that the EO extracted from *A. annua* had an antioxidant activity equivalent to 18% of the positive control (α-tocopherol) [30].

Several studies have estimated the antioxidant character of the *Artemisia* genus EO from different species, and the current study of the (AJ) EO showed the strongest antioxidant potential compared with these studies [30,31,32,33].

The antioxidant character of (AJ) EO may be attributed to the presence of a high percentage of bornyl acetate which also showed potential antioxidant activity in several published works and approved that it has the potentials to scavenge free radicals and reduce oxidative stress [34,35,36].

### 3.3. Metabolic Lipase, α-Amylase, and α-Glucosidase Inhibitory Activities

Obesity, overweightness, diabetes, and dyslipidemia are global health problems with increasing prevalence [37]. Many scientific reports have found that they are directly associated with an increase in mortality rates of cancer and cardiovascular, respiratory, hepatic, and renal diseases [38]. In the last three decades, in Palestine and many other countries, the high prevalence of these metabolic disorders has been obvious and worrisome [39]. In fact, any pharmacological agent that can inhibit the actions of the vital metabolic enzymes, including lipase, α-amylase, and α-glucosidase, can inevitably cure these life-threatening disorders [40]. Actually, in various ethnopharmacological global systems of medicine, aromatic plants have long been utilized for the treatment of obesity, overweightness, dyslipidemia, and diabetes [41,42].

In the current study, the results showed that the (AJ) EO exhibited weak lipase inhibitory potential at the tested concentrations of 50, 100, 200, 300, and 400 μg/mL, with inhibition in a dose-dependent manner and an IC_50_ value of 51.41 ± 0.91 μg/mL; although, the anti-lipase IC_50_ potential of the positive control (orlistat) was 0.13 ± 0.86 μg/mL.

Among many ethnomedicinal plants, many *Artemisia* species are traditionally utilized for the treatment of diabetes in several folk medicine systems [43,44]. Therefore, the current investigation was focused on assessing the ability of the (AJ) EO to inhibit the target carbohydrate metabolic enzyme inhibitors, including α-amylase and α-glucosidase. The results showed that the tested EO exhibited α-amylase suppressing effect compared with the positive control, acarbose, with IC_50_ values of 14.17 ± 0.39 and 8.53 ± 0.72 μg/mL, respectively, at the tested concentrations (10, 50, 70, 100, and 500 μg/mL). Notably, the (AJ) EO exhibited strong α-glucosidase inhibitory potential at the tested concentrations (100, 200, 300, 400, and 500 mg/mL), with inhibition in a concentration-dependent manner, compared with the positive control, acarbose, with IC_50_ values of 144.45 ± 0.88 and 62.36 ± 1.05 μg/mL, respectively.

Olennikov et al. investigated the α-amylase and α-glucosidase inhibitory activities of 12 Artemisia species which included A. umbrosa, A. tanacetifolia, A. sericea, A. palustris, A. messerschmidtiana, A. macrocephala, A. leucophylla, A. latifolia, A. integrifolia, A. desertorum, A. commutata, and A. anethifolia and found that these species had an α-glucosidase inhibitory activity with IC_50_ range 214.42–754.12 µg/mL and α-amylase suppressant potential with IC_50_ range of 150.24–384.14 µg/mL) [45].

The current study is the first investigation that has assessed the inhibitory characteristics of the (AJ) EOs against lipase, α-amylase, and α-glucosidase.

### 3.4. Cytotoxicity

To understand the effect of the (AJ) EO on human cancer cells, the current investigation was carried out utilizing cultured HeLa, Caco-2, MCF-7, and Hep3B tumor cell lines. Viability results were measured using the trypan blue MTS assay. The results revealed that incubation of tumor cells with the (AJ) EO (1.25, 0.625, 0.3125, 0.15625, and 0.078125 mg/mL) for 24 h reduced the viability of these cells in a dose-dependent manner. The number of dead cells increased by increasing the concentration of the (AJ) EO. The highest cytotoxic effect was observed against MCF-7 followed by Caco-2, Hep3B and HeLa cancer cells, with IC_50_ values of 255 ± 2.11, 379.12 ± 1.98, 440.12 ± 3.11 and 15,412 ± 2.2 µg/mL, respectively, compared with the potential anticancer drug doxorubicin (positive control), which had cytotoxic IC_50_ values of 0.43 ± 0.06, 0.37 ± 0.08, 1.21 ± 0.05, and 0.84 ± 0.03 µg/mL, respectively.

Bornyl acetate the chief constituent in the current study had a cytotoxic effect compared with the cis-platin anticancer drug with IC_50_ values of 71.97 and 126.75, respectively.

### 3.5. Antimicrobial Activity

Microbial resistance has become one of the most major global challenges during the last two decades, referring primarily to the overuse or misuse of antibiotics. Every year, approximately 2 million people in the United States are infected with antibiotic-resistant bacteria. Microbial resistance kills 700,000 people every year around the world [46].

Many EO-bearing plants have been utilized for the treatment of many infectious illnesses since ancient times. Indeed, EOs played an essential role in the design of novel potential bacteriostatic and fungistatic drugs such as thymol, eugenol, caryophyllene, and many others [47].

The EO from the dried leaves of (AJ) showed potent antimicrobial properties with higher antibacterial activity against MRSA, *S. aureus*, and *P. vulgaris*, each showing a MIC of 0.625 μg/mL than the commercial antibiotic ciprofloxacin which has a MIC of 12.5, 0.78, and 15 μg/mL, respectively. The EO also showed more potent antibacterial activity than ampicillin which does not have antibacterial activity against MRSA and has weak antibacterial activity against the rest of the bacterial strains. Indeed, the EO did not show any inhibitory activity against the growth of *E. coli* and *P. aeruginosa* strains.

Interestingly, the (AJ) EO showed a ten-fold more potent antifungal activity against *C. albicans* than the commercial anticandidal drug fluconazole, with a MIC of 0.156 and 1.56 μg/mL, respectively. There was no noticeable difference in sensitivity between Gram-positive and Gram-negative bacteria, while it is understood that the Gram-negative bacteria are less susceptible to EOs due to the existence of hydrophilic lipopolysaccharides (LPS) in their outer membrane. LPS prevents various hydrophobic EO compounds from penetrating the membrane. Actually, EOs and their hydrophobic ingredients can destroy bacterial cell membranes by removing their lipid fraction, impairing the bacterial cell’s ability to survive [48,49,50].

EOs from different *Artemisia* species are well documented for their antimicrobial activity and several investigations have reported that they displayed remarkable antibacterial and antifungal activities against several microbial strains [51,52,53].

The results of the present investigation revealed that (AJ) EO has potent antibacterial and antifungal potentials against certain strains of microbial species. Actually, bornyl acetate, the study’s main molecule, has long been recognized as a potent antimicrobial agent, according to numerous published studies [36,54,55].

### 3.6. Cyclooxygenase Inhibitory Activity

Cyclooxygenase inhibitory activity is an important tool to control the inflammation process in the human body. In fact, inflammation is an important physiological response of autoimmune activation, infection, or cellular injury. The excess production of proinflammatory mediators may cause different kinds of severe and chronic inflammation that lead to several illnesses including atherosclerosis, multiple sclerosis, asthma, arthritis, and rheumatoid arthritis [56].

The (AJ) EO COX inhibition activity test showed that the EO has more selective inhibition towards COX-1. Its COX-2 selectivity value hence was lower (0.17) and comparable to that of ketoprofen (0.196). The percentage inhibition of COX-2 increased with an increase in the concentration of EO used. When the EO concentration was increased from 50 to 350 µg/mL, inhibition increased from 42.9% to 65.3%. Similarly, COX-1 inhibition also increased with increasing EO concentration. Increasing the EO concentration from 50 to 350 µg/mL caused an increase in COX-1 inhibition from 57.2% to 68.2%.

The current study results showed better inhibition activity of the extracted (AJ) EO as 50 µg/mL caused 57.2% and 42.5% inhibition of COX-1 and COX-2, respectively. However, the (AJ) EO has more selectivity towards COX-1 hence its COX-2 inhibition selectivity < 1 (0.017) compared with that of the commercial pharmaceutical preparation celecoxib [57]. The EO of (AJ) inhibited the enzymes COX-1 and COX-2 with IC_50_ values of 15.64 and 91.91 µg/mL, respectively, compared with the pharmaceutical anti-inflammatory drug ketoprofen which has an IC_50_ of 7.89 and 40.18 µg/mL, respectively.

Up to the knowledge of the authors, the current investigation represents the first phytochemical and biological experimental works carried out on the (AJ) plant EO and consequently, it might create the basis for more in vivo trials in search of eco-friendly green medications.

## 4. Material and Methods

### 4.1. Collection and Drying of the Plant

In July 2020, the leaves of the (AJ) plant were collected in Hebron’s southern area, near Palestine’s Al-Naqab desert. Dr. Nidal Jaradat, a pharmacognosist at An-Najah National University’s Pharmacy Department, described the plant, and the voucher specimen was stored in the Herbal Products Laboratory (Pharm-PCT-237). The collected materials were cleaned and dried at ordinary room temperature (25 ± 4 °C) and humidity (50 ± 6% RH) in the shade for 17 days. The dried leaves were then coarsely ground and kept in glass jars for further use.

### 4.2. Isolation of Artemisia jordanica Essential Oil

The EOs of the (AJ) plant were separated, utilizing the hydro-distillation procedure pronounced by Jaradat et al. [58]. Briefly, 0.1 kg of the dried leaf powder was suspended with 1 L of distilled water, and the EO was extracted at 100 °C using a Clevenger device (Deschem, Changshu, China) operating at atmospheric pressure for 180 min. The obtained (AJ) EO was chemically dried utilizing calcium carbonate and stored at 2 °C in a refrigerator until further use. The extracted EO yield was 1.31% *v*/*w*.

### 4.3. Characterization of Artemisia jordanica Essential Oil

The phytochemical profile of A (AJ) EO was determined utilizing a gas chromatograph (Hewlett Packard HP 5890 Series II, Houston, TX, USA) connected with mass spectrometry (PerkinElmer, Elite-5-MS, Massachusetts, United States) with a fused-silica capillary column (30 m × 0.25 mm, with a film thickness of 0.25 µm). at a flow rate of 1.1 mL/min, the helium gas was set while the injector temperature was fixed at 250 °C, the oven temperature was set at 50 °C for 5 min followed by a ramp of 4.0 °C/min to 280 °C. The total running time was 62.50 min, and the solvent delay was from 0 to 4.0 min. The mass spectroscopy (MS) scan time was from 4 to 62.5 min, covering a mass range of 50.00 to 300.00 m/z. The mass spectra were collected under electronic ionization conditions at 70 eV [59]. In brief, retention indices (RIs) have been calculated according to the injected standard mixture of normal alkanes (C6–C27) under the mentioned conditions using the following well-known equation approved by the International Union of Pure and Applied Chemistry (IUPAC) (https://goldbook.iupac.org/terms/view/R05360). The identification was also confirmed by comparison of their mass spectra with those stored in the Wiley7n.l MS computer library. The linear temperature-programmed RIs of all the constituents were calculated from the gas chromatogram by interpolation between bracketing *n*-alkanes using the following equation:

RI = 100 × (((tR(i) − tR(z))/(tR(z + 1) − tR(z))) + z)(1)

where z is the number of carbon atoms in the smaller n-alkane and tR(i), tR(z), and tR(z+1) are the retention times of the desired compound, the smaller n-alkane, and the larger n-alkane, respectively.

### 4.4. Free Radical Scavenging Activity

One hundred mg of (AJ) EO was dissolved in 100 mL of methanol to create the stock solution (1 mg/mL). The obtained solution was mixed with methanol to obtain different concentrations (2, 3, 5, 7, 10, 20, 30, 40, 50, 80, and 100 μg/mL). One milliliter of stock solution and 1 mL of methanol were mixed with 1 mL of DPPH (Sigma-Aldrich, Steinheim, Germany). Then, the solution was kept in the dark at room temperature for 30 min. However, by replacing the plant EO solution with methanol, the blank solution was prepared. Trolox (Sigma-Aldrich, St. Louis, MO, USA) was utilized as a positive control, and the absorbance for all obtained samples was measured by spectrophotometer (Shimadzu-UV-1800, Kyoto, Japan) at 517 nm. The DPPH inhibitory potentials were estimated utilizing the following formula:

I (%) = [ABS_blank_ − ABS_test_]/[ABS_blank_]) × 100% (2)

where I (%) is the percentage of DPPH inhibitory potentials [60,61].

### 4.5. Porcine Pancreatic Lipase Inhibitory Activity

A working solution (1 mg/mL) was done by dissolving 100 mg of the (AJ) EO in 100 mL of 10% dimethyl sulfoxide (DMSO) (Riedeldehan, Seelze, Germany). Then, the obtained solution was diluted to produce the following concentrations: 400, 300, 200, 100, and 50 μg/mL. Porcine pancreatic lipase enzyme (Sigma, St. Louis,, MO, USA) stock solution (1 mg/mL) was directly prepared before use by dissolving 25 mg of lipase enzyme powder in 25 mL of 10% DMSO. Then, *p*-nitrophenyl butyrate (*P*NPB) (Sigma-Aldrich, Schnelldorf, Germany) stock solution was prepared by dissolving 20.9 mg of *P*NPB in 2 mL of acetonitrile. From the prepared serial dilutions of the (AJ) EO, 0.2 mL was mixed with 0.1 mL of the lipase enzyme stock solution and tris-HCl (Sigma, MO, USA) to reach a volume of 1 mL. Then, the solution was incubated for 15 min at 37 °C in a water bath. After 15 min, 100 μL of *P*NPB solution was added, and the solution was incubated for 30 min at 37 °C. A 1 mL blank solution was prepared by mixing 100 μL of lipase enzyme (1 mg/mL) solution with a tris-HCl solution. The commercial anti-obesity drug orlistat (Sigma-Aldrich, Schnelldorf, Germany) was utilized as a positive control, and we followed the same previous steps as the (AJ) EO. The absorbance was measured utilizing a UV–Vis-spectrophotometer at 405 nm. However, the lipase enzyme inhibitory potential was measured utilizing the following equation:

I (%) = [ABS_blank_ − ABS_test_]/[ABS_blank_]) × 100% (3)

where I (%) is the percent inhibition of the lipase enzyme [62].

### 4.6. α-Amylase Inhibitory Activity

A stock solution (1 mg/mL) was prepared by dissolving 25 mg of the (AJ) EO in a small amount of 10% DMSO. Then, a buffer solution was added to 25 mL. The solution was then diluted with the buffer to obtain different dilutions (10, 50, 70, 100, and 500 μg/mL). Later on, the porcine pancreatic α-amylase enzyme (Sigma-Aldrich, USA) stock solution (2 units/mL) was made by mixing 12.5 mg of α-amylase powder with a minimum amount of 10% DMSO, and the volume was completed with a buffer solution to 100 mL. Then, corn starch (Alzahraa company, Palestine) solution was prepared by dissolving 1 g of starch in 100 mL of distilled water. Two hundred microliters of the (AJ) EO stock solution were mixed with 200 μL of the α-amylase stock solution, and the solution was incubated for 10 min at 30 °C in a water bath. After that, 200 μL of corn starch solution was added and incubated for 3 min at 30 °C. Moreover, 3,5-dinitrosalicylic acid (Sigma, India) was added and boiled in a water bath at 85–90 °C for 10 min, and after the solution was cooled, 5 mL of distilled water was added. The blank solution was prepared by replacing the (AJ) plant EO with 200 μL of a buffer solution. Acarbose was used as a positive reference compound while the absorbance was assessed at 540 nm using a UV–Vis- spectrophotometer. The α-amylase inhibitory potential was calculated by the following formula:

I (%) = [ABS_blank_ − ABS_test_]/[ABS_blank_] × 100% (4)

where I (%)  is the α-amylase inhibitory percentage [63,64].

### 4.7. α-Glucosidase Inhibitory Activity

The α-glucosidase inhibitory activity of the (AJ) EO was determined according to the standard protocol, with some modifications [40]. In each test tube, a reaction mixture containing 50 μL of phosphate buffer (100 mM, pH = 6. 8), 10 μL of α-glucosidase (1 U/mL), and 20 μL of varying concentrations of (AJ) EO (100, 200, 300, 400, and 500 μg/mL) was incubated at 37 °C for 15 min. Then, the preincubated 20 μL of (5 mM) *P*NPG (4-Nitrophenyl-β-D-glucopyranoside) was added as a substrate of the reaction and was again incubated at 37 °C for an additional 20 min. The reaction was terminated by adding 50 μL of Na_2_CO_3_ (0.1 M). The absorbance of the released p-nitrophenol was measured by a UV–Vis spectrophotometer at 405 nm. With similar concentrations as the plant EO, acarbose was used as a positive control.

The inhibition percentage of the (AJ) EO was calculated using the following equation:

(5) $$\alpha -\mathrm{Glucosidase}\;\%=\frac{A_{b}-A_{s}}{A_{b}}\times 100$$

where *A_b_* is the absorbance of the blank and *A_S_* is the absorbance of the tested sample or control.

### 4.8. Cell Culture and Cytotoxicity Assay

The liver (Hep3B), breast (MCF-7), Human cervical (HeLa), and Colon (CACO-2) tumor cell lines were cultivated separately in RPMI-1640 media (Sigma, Norwich, UK), which was treated with 1% *L*-glutamine (Sigma, London, UK), 1% penicillin/streptomycin antibiotics (BI, New Delhi, India), and 10% fetal bovine serum. Cancer cells were grown at 37 °C in a humidified atmosphere with 5% CO_2_. Cells were seeded at 2.6 × 10^4^ cells/well in a 96-well plate. After 48 h, cancer cells were incubated with various concentrations (500, 120, 60, 30, and 10 µg/mL) of the (AJ) EO and doxorubicin (positive control) for 24 h. Cell viability was evaluated by the CellTilter 96^®^ Aqueous One Solution Cell Proliferation (MTS) Assay according to the manufacturer’s directions (Promega Corporation, Madison, WI, USA). At the end of the treatment, 20 μL of MTS solution per 100 μL of media was added to each well, and the solutions in the well plates were incubated at 37 °C for 2 h. The absorbance was measured at 490 nm [65,66].

### 4.9. Microbial Strains, Culture Media, and Antimicrobial Assay

The antibacterial effect of (AJ) EO was determined utilizing several strains of bacteria, which were obtained from the American Type Culture Collection (ATCC): *Pseudomonas aeruginosa* (ATCC 9027), *Escherichia coli* (ATCC 25922), *Klebsiella pneumonia* (ATCC 13883), *Proteus vulgaris* (ATCC 8427), and *Staphylococcus aureus* (ATCC 25923), and from a diagnostically confirmed methicillin-resistant *Staphylococcus aureus* (MRSA). The antifungal activity of (AJ) EO was evaluated against the growth of *Candida albicans* (ATCC 90028). The antimicrobial activity of the (AJ) EO used in this study was estimated using the broth microdilution method. The (AJ) EO was dissolved in DMSO to a concentration of 200 µg/mL. The produced solution was serially micro-diluted (2-fold) 10 times in sterile Mueller–Hinton broth (Thermo Fisher Scientific, Waltham, MA, USA). The dilution processes were performed under aseptic conditions in 96-well plates. In the micro-wells that were assigned to evaluate the antibacterial activity of (AJ) EO, micro-well number 11 contained plant-free Mueller–Hinton broth, which was used as a positive control for microbial growth. Micro-well number 12 contained plant-free and microbial-free Mueller–Hinton broth, which was used as a negative control for microbial growth. Micro-wells numbered 1–11 were inoculated aseptically with the test microbes. The (AJ) EO antimicrobial activity was determined in triplicate. All the inoculated plates were incubated at 35 °C. Regarding *C. albicans*, the same method was used but using RPMI media (Thermo Fisher Scientific, Waltham, MA, USA) instead of Mueller–Hinton broth. The incubation period lasted for about 18–24 h for those plates inoculated with the test bacterial strains and for about 48 h for those plates inoculated with *C. albicans*. The lowest concentration of (AJ) EO at which no visible microbial growth was observed in the micro-well was considered as the minimum inhibitory concentration (MIC) of the examined EO. Antimicrobial activity was evaluated using known antimicrobial agents, namely ampicillin and ciprofloxacin, which were used as positive controls for antibacterial activity, and fluconazole, which was used as the positive control for antifungal activity [67,68].

### 4.10. COX Inhibitory Assay

The ability of the (AJ) EO to prevent the conversion of arachidonic acid to PGH2 by bovine COX-1 and human recombinant COX-2 was assessed using a COX inhibitor screening assay kit (Item No: 560131, Cayman Chemical, Ann Arbor, MI, USA) according to manufacturer’s guidelines. The 50% inhibitory concentration (IC_50_) of COX-1/COX-2 activity of the tested samples was determined, with the assay run, in duplicate, with two concentrations (350 and 50 µg/mL). A standard curve of eight concentrations of prostaglandin, a non-specific binding sample, and a maximum binding sample was used, as instructed in the kit manual, to determine the inhibition of the sample plant, applying the generated multiple regression best-fit line. The percentage inhibition of the two concentrations was used to calculate the IC_50_ [69,70].

### 4.11. Statistical Analysis

All of the (AJ) EO’s antioxidant, antimicrobial, antilipase, cytotoxicity, COX, -amylase, and -glucosidase assays were carried out in triplicate, and the results were presented as means ± SD. Differences were considered to be significant when p values were lower than 0.05. The SPSS program, Version 22.0 for Windows was used for all statistical analyses (IBM SPSS Inc., Chicago, IL, USA).

## 5. Conclusions

Overall, the current investigation is the first one that explored the chemical constituents, antioxidant, antimicrobial, vital physiological enzymes inhibitory and cytotoxic effects of (AJ) EO. The results revealed that oxygenated terpenoids including bornyl acetate and endo-borneol presented are the major components of the (AJ) EO. Compared with the used positive controls, the (AJ) EO has strong antioxidant, antibacterial, antifungal, anti-α-amylase, anti-α-glucosidase, and COX inhibitory effects which could be a favorite candidate for the treatment of oxidative stress, microbial resistance, diabetes mellitus, and inflammatory diseases. Preclinical and clinical investigations are required to be conducted on the use of this plant species and further in-depth investigations are urgently crucial to explore the importance of such medicinal plants in pharmaceutical production.

## Figures and Tables

**Table 1:** The chemical ingredients of *Artemisia jordanica* essential oil.

Name	Retention Index_calculated_	Retention Index_Litreture_ [22]	Essential Oil (%)
Thujene	912	911	0.13
a-Pinene	936	936	0.28
Camphene	953	954	3.62
Sabinene	979	979	0.26
2,3-Dehydro-1,8-Cineole	991	991	3.56
Cineole	1040	1041	0.20
Camphor	1142	1141	0.57
Nirol oxide	1161	1160	0.23
Terpinen-4-ol	1163	1162	0.27
Endo-borneol	1178	1178	17.75
Bornyl acetate	1280	1280	63.40
Caryophyllene	1420	1421	1.43
Germacrene	1489	1489	0.14
Caryophyllene oxide	1574	1574	0.14
Geranyl isovalerate	1602	1602	7.67
14-Hydroxy-(Z)-caryophelene	1667	1667	0.07
Germacra-4(15),5,10(14)-trien-1α-ol	1674	1674	0.14
Farnesyl	1803	1803	0.10
**Phytochemical Classes**			
Monoterpene hydrocarbon			4.28
Oxygenated monoterpenoid			85.98
Sesquiterpene hydrocarbon			1.66
Oxygenated sesquiterpenoid			8.01

**Table 2:** The IC_50_ (µg/mL) for *Artemisia jordanica* essential oil against DPPH, lipase, α-amylase, α-glucosidase, and cancer cells in comparison with the positive controls.

Antioxidant, Target Metabolic Enzymes, and Cancer Cells Lines	IC_50_ (µg/mL)	
	*Artemisia jordanica* Essential Oil	Positive Controls
**DPPH**	2.18 ± 0.24	1.58 ± 1.49 ^a^
**Lipase**	51.41 ± 0.91	0.13 ± 0.86 ^b^
**α-Amylase**	14.17 ± 0.39	8.53 ± 0.72 ^c^
**α-Glucosidase**	144.45 ± 0.88	62.36 ± 1.05 ^c^
**Caco-2**	379.12 ± 1.98	0.37 ± 0.08 ^d^
**HeLa**	15412 ± 2.2	0.84 ± 0.03 ^d^
**MCF7**	2550 ± 2.11	0.43 ± 0.06 ^d^
**HepG2**	440.12 ± 3.11	1.21 ± 0.05 ^d^

^a^ trolox; ^b^ orlistat; ^c^ acarbose; ^d^ doxorubicin.

**Table 3:** MIC values (µg/mL) of *Artemisia jordanica* essential oil, ampicillin, ciprofloxacin and fluconazole.

Tested Samples	Microbial Strains						
	MRSA	*S. aureus*	*E. coli*	*K. pneumoniae*	*P. vulgaris*	*P. aeruginosa*	*C. albicans*
Fluconazole	−	−	−	−	−	−	1.56
Ampicillin	R	3.12	3.12	1	18	R	−
Ciprofloxacin	12.5	0.78	1.56	0.125	15	3.12	−
*Artemisia jordanica* essential oil	0.625	0.625	R	2.5	0.625	R	0.156

R: Resistant.

**Table 4:** IC_50_ values of COX-1 and COX-2 and COX-2 inhibition selectivity of *Artemisia jordanica* essential oil.

Name	IC_50_ (µg/mL)		Selectivity Ratio for COX-2
	COX-1	COX-2	
Ketoprofen	7.89 ± 0.96	40.18 ± 1.09	0.196
*Artemisia jordanica* essential oil	15.64 ± 0.67	91.91 ± 1.91	0.170

## Data Availability

All data is contained within the article.

## Sample Availability

Sample of the *Artemisia jordanica* essential oil are available from the authors.
